# Rapid cell culture and pre-clinical screening of a transforming growth factor-β (TGF-β) inhibitor for orthopaedics

**DOI:** 10.1186/1471-2474-11-105

**Published:** 2010-05-28

**Authors:** Aaron Schindeler, Alyson Morse, Lauren Peacock, Kathy Mikulec, Nicole YC Yu, Renjing Liu, Sandy Kijumnuayporn, Michelle M McDonald, Paul A Baldock, Andrew J Ruys, David G Little

**Affiliations:** 1 Department of Orthopaedic Research & Biotechnology, The Children's Hospital at Westmead, Locked Bag 4001, Westmead, NSW 2145, Australia; 2 Discipline of Paediatrics and Child Health, Faculty of Medicine, A27 University of Sydney, NSW 2006, Australia; 3 School of Aerospace, Mechanical and Mechatronic Engineering, J07 University of Sydney, NSW 2006, Australia; 4 Osteoporosis and Bone Biology Research Program, Garvan Institute of Medical Research, 384 Victoria St, Darlinghurst, NSW 2010, Australia

## Abstract

**Background:**

Transforming growth factor-β (TGF-β) and bone morphogenetic proteins (BMPs) utilize parallel and related signaling pathways, however the interaction between these pathways in bone remains unclear. TGF-β inhibition has been previously reported to promote osteogenic differentiation *in vitro*, suggesting it may have a capacity to augment orthopaedic repair. We have explored this concept using an approach that represents a template for the testing of agents with prospective orthopaedic applications.

**Methods:**

The effects of BMP-2, TGF-β1, and the TGF-β receptor (ALK-4/5/7) inhibitor SB431542 on osteogenic differentiation were tested in the MC3T3-E1 murine pre-osteoblast cell line. Outcome measures included alkaline phosphatase staining, matrix mineralization, osteogenic gene expression (*Runx2, Alp, Ocn*) and phosphorylation of SMAD transcription factors. Next we examined the effects of SB431542 in two orthopaedic animal models. The first was a marrow ablation model where reaming of the femur leads to new intramedullary bone formation. In a second model, 20 μg rhBMP-2 in a polymer carrier was surgically introduced to the hind limb musculature to produce ectopic bone nodules.

**Results:**

BMP-2 and SB431542 increased the expression of osteogenic markers *in vitro*, while TGF-β1 decreased their expression. Both BMP-2 and SB431542 were found to stimulate pSMAD1 and we also observed a non-canonical repression of pSMAD2. In contrast, neither *in vivo *system was able to provide evidence of improved bone formation or repair with SB431542 treatment. In the marrow ablation model, systemic dosing with up to 10 mg/kg/day SB431542 did not significantly increase reaming-induced bone formation compared to vehicle only controls. In the ectopic bone model, local co-administration of 38 μg or 192 μg SB431542 did not increase bone formation.

**Conclusions:**

ALK-4/5/7 inhibitors can promote osteogenic differentiation *in vitro*, but this may not readily translate to *in vivo *orthopaedic applications.

## Background

Bone Morphogenetic Proteins (BMPs) are critical in the formation of cartilage and bone. Osteogenic BMPs, such as BMP-2, and -7 are widely recognized to promote an osteogenic response [1]. Transforming Growth Factor-β (TGF-β) belongs to the same superfamily as the BMPs, although its role in bone is less clear. Both BMPs and TGF-β bind to related Type I receptors (also known as Activin Receptor-like Kinases or ALKs) and Type II receptors and activate downstream SMAD signaling pathways [2-4]. The osteogenic BMPs can bind to ALKs 1/2/3/6 and BMPRII or ActRII to induce phosphorylation of the receptor-regulated SMADs (R-SMADs) 1/5/8. In the case of TGF-β and the non-osteogenic BMPs, ligand binding to receptors such as ALKs 4/5/7 and TβRII induces phosphorylation of R-SMADs 2/3.

There is conflicting evidence on the effects of TGF-β signaling on bone formation. TGF-β isoforms are robustly expressed during the early stages of bone healing [5,6], and exogenous TGF-β has been purported to augment bone markers in cultured human osteoblasts [7] and can lead to improvements in bone repair in orthopaedic animal models [8-10]. However, in cultured murine cell lines, TGF-β acting through SMAD3 was reported to antagonize osteogenesis [11,12] and comparable findings were found in human mesenchymal stem cells [13]. Further work suggests that exogenous TGF-β can delay osteogenesis in favor of chondrogenesis [14]. In addition to direct effects on osteogenic differentiation, TGF-β may also lead to increased fibrosis. In rodent distraction osteogenesis and fracture models, TGF-β1 and TGF-β2 treatment (respectively) did not lead to improved outcomes but did result in increased fibrous and cartilage tissue [15,16]. In these studies, inflammation and edema were also reported as unfavorable side-effects.

TGF-β signaling has also been linked to other fibrotic conditions, such as the genetic disorder Marfan syndrome. Animal models with aberrant TGF-β signaling have been successfully treated with TGF-β neutralizing antibody or with Losartan, a small-molecule angiotensin II AT_1 _receptor blocker (ARB) [17-19]. ARBs are now under trial for Marfans syndrome [20], and may be applicable for other TGF-β related disorders. However, the affects of ARBs on TGF-β protein expression are indirect and do not appear to translate to bone [21], thus making these agents less attractive for orthopaedic applications. In contrast, a novel synthetic compound, SB431542, has been shown to rapidly and selectively inhibit ALK-4/5/7 but not ALK-2/3/6 kinase activity [22]. This enables the blockade of the classical TGF-β-SMAD2/3 signaling pathway whilst allowing osteogenic BMP-SMAD1/5/8 signaling.

In a seminal study by Maeda *et al*. (2004), SB431542 repression of TGF-β signaling was found to enhance osteoblastic differentiation in BMP-2 treated C2C12 myoblasts [23]. Osteoblastic differentiation and matrix mineralization were also increased in cultured human mesenchymal stem cells. Based on these *in vitro *findings, we speculated that this compound may also be able to positively influence bone formation or healing. As a putative anti-fibrotic agent, SB431542 could have additional benefits in the context of orthopaedic repair where fibrosis is problematic.

In this study we have used both *in vitro *and *in vivo *methods suitable for the rapid screening of compounds specifically for orthopaedic applications. These assays represent a systematic approach that can be readily applied to other putative pro-osteogenic agents. In cell culture experiments, we treated the MC3T3-E1 pre-osteoblast cell line with purified recombinant BMP-2, purified TGF-β1, and the TGF-β receptor inhibitor SB431542, individually and in combination. Outcome measures included alkaline phosphatase (AP) and mineralization staining, osteogenic gene expression, and activation of downstream SMAD signaling pathways. Next, we attempted to translate the effects of TGF-β inhibition using animal models. This included a marrow ablation model (where intramedullary reaming produces bone formation over a 10-day period via intramembranous ossification), and BMP-2 implantation (where ectopic bone nodules are induced in muscle over 3 weeks via endochondral ossification). This study design represents a straightforward methodology for testing prospective orthopaedic agents.

## Methods

### Cell culture methods

MC3T3-E1 pre-osteoblasts were grown in α-MEM media containing 10% FBS (Invitrogen, Carlsbad, CA, USA). Passage number 20 cells were used, and cultured for no more than 2 weeks prior to initiating differentiation. Osteogenic differentiation was instigated by supplementing media with 50 mg/L ascorbic acid and 10 mM β-glycerophosphate (Sigma Aldrich, St Louis, MO, USA). All culture media contained 2 mM L-glutamine, and antibiotics (100 units/ml penicillin and 0.1 mg/ml streptomycin) (Invitrogen). Cultures were grown in 37°C incubators at 5% CO_2 _with media changes every 2-3 days. For staining experiments cells were plated in 48-well plates at 5 × 10^4 ^cells/well. For cDNA or protein collection experiments cells were seeded in 6-well plates at 2 × 10^5 ^cells/well. Cells were plated overnight and were sub-confluent prior to the addition of drugs or recombinant proteins.

### Recombinant proteins and drugs

Recombinant human BMP-2 (rhBMP-2) (Medtronic Australasia, North Ryde, NSW, Australia) was solubilized in sterile water at stock concentrations of 100 μg/ml. Transforming growth factor-beta 1 (TGF-β1) was purchased from Sigma Aldrich (T1654), and reconstituted at 1 μg/ml in filtered 0.1% BSA in 4 mM HCl. The ALK-4/5/7 inhibitor SB431542 was purchased from Sigma Aldrich (S4317) and solubilized in dimethylsulfoxide (DMSO) at stock concentration of 10 mM. For *in vitro *experiments, all wells received the same volume of DMSO (<0.1%) to avoid the confounding effects of DMSO on osteogenic differentiation [24]. PTH_(1-34) _peptide was purchased from Auspep (Parkville, VIC, USA).

### Alkaline phosphatase (AP) and Alizarin Red S staining

Prior to staining, all cells were washed with PBS and fixed with 4% paraformaldehyde (PFA). AP staining was performed using a solution containing 0.5 mg/ml naphthol AS-BI phosphate (Sigma Aldrich), 0.5 mg/ml Fast Blue (Sigma Aldrich), 10% N,N-dimethylformamide (Sigma Aldrich), 0.5% MgCl_2 _in 0.1 M Tris (pH 9.4) for 10 min at room temperature [25]. Mineralization of calcium deposits was assessed by Alizarin Red S staining (40 mM, pH 4.2) (LabChem, Pittsburgh, PA, USA) for 10 min at room temperature, followed by multiple washes with distilled water to remove background staining [26].

#### RNA extraction, cDNA preparation, and real-time quantitative PCR (qPCR)

Total RNA was isolated using TRIZOL reagent (Invitrogen). Equivalent amounts of total RNA were used to synthesize cDNA by reverse-transcription using Superscript III Reverse Transcriptase (Invitrogen). All samples were amplified using the SYBR Green PCR reagent kit (Integrated Sciences, Chatswood, NSW, Australia). PCR was performed on the Rotor-Gene 3000 (Corbett Life Science, Sydney, NSW, Australia). PCR primer sequences and amplification conditions are as previously published [27]. PCR reactions were performed in quadruplicate and normalized to the housekeeping *Gapdh*. Data are presented as mean fold induction relative to untreated cells, with the standard deviation shown in parentheses.

#### Western blotting

Cell extracts were harvested in SDS sample buffer, denatured at 95°C for 10 min, separated by electrophoresis on a 1% SDS-PAGE gel, and then transferred by semi-dry electroblotting to PVDF membrane (Millipore, Billerica, MA, USA) [27]. Immunoblotting was performed with anti-Smad1 and anti-Smad2 (Zymed/Invitrogen), anti-pSmad1 and anti-pSmad2 (Cell Signaling Technology, Boston, MA, USA), and α-Tubulin (Sigma-Aldrich) antibodies. Horseradish peroxidase-conjugated donkey anti-rabbit IgG and sheep anti-mouse IgG secondary antibodies were used (GE Healthcare, Little Chalfont, Buckinghamshire, UK). Chemiluminescence reagents (New England Biolabs, Ipswich, MA, USA) were used to visualize the blots on X-ray film.

#### Surgical models

Experiments using mice were approved by the SWAHS Animal Ethics Committee (Protocol 5031). In all studies, 8-10 week old C57BL6/J mice were used and anesthesia was induced with inhaled isofluorane. Pain was managed using buprenorphine (0.05-0.1 mg/kg) subcutaneously (s.c.) postoperatively (then every 12 hours as required). Dehydration was managed by saline injection as required.

##### Marrow Ablation Model

New intramedullary bone was induced by femoral reaming as previously described in rats [28] and mice [29]. An incision was made on the right hind leg lateral to the knee and a 25G needle pushed through the cortex of the distal femur between the intercondyles and unilaterally up the medullary canal to loosen the marrow. This needle was removed and 0.1 ml of saline was injected into the cavity to flush out the marrow using a 30G needle. The wound was closed using 5-0 nylon suture (Ethicon). Mice were dosed s.c. daily with SB431542 in DMSO from the day of surgery until sacrifice at day 10. Control groups received an equivalent volume of vehicle (DMSO) or the bone inducing agent 25 μg/kg/day PTH_1-34 _(Auspep). In an initial study, 1 mg/kg/day SB43152, DMSO control, and PTH_1-34 _groups were examined (N = 6). In a second study, a wider dose range was used of 0.1 mg/kg/day, 1 mg/kg/day, and 10 mg/kg/day SB431542 (N = 6) with DMSO controls (N = 6) and a nominal number of repeated PTH_(1-34) _controls (N = 2).

##### BMP-2 Implantation Model

New bone formation in a quadriceps muscle pouch was assayed after 3 weeks following the implantation of a BMP-2 containing pellet. Pellets containing 20 μg recombinant human BMP-2 (Medronic Australasia) and 0 μg, 38 μg, or 192 μg SB431542 (Sigma-Aldrich) were manufactured by solubilizing Poly-(D, L-lactic acid) polymer (viscosity 0.55-0.75, average M_w _75000-12000, Sigma-Aldrich) and drugs in ethyl acetate (analytical reagent grade, Chem Supply, Gillman, SA, Australia) and evaporating the solvent under vacuum. The BMP-2 dose was previously determined empirically using this polymer delivery system and the ratio of BMP-2/SB431542 was based on *in vitro *efficacy. Pellets were molded by compression in a modified 0.3 ml syringe. Pellets were surgically implanted by a surgeon experienced in mice using published methods [25]. Group sizes of N = 8 were used.

### Radiographic analysis

The bone formed in both models was examined at the experimental endpoint using a digital X-ray machine (Faxitron X-ray Corp., Wheeling, IL, United States).

To quantify the total bone mineral content (BMC) in the distal region of reamed and unreamed femora, peripheral Quantitative Computed Tomography (pQCT) and analysis was performed using a XCT-960A scanner (Stratec Medizintechnik, Pforzheim, Germany). A region spanning the midshaft to the distal femur was selected after a pre-scan and measurements were taken from ten 2 × 5 × 0.2 mm slices using a red collimation mask. These values were averaged and for each mouse the reaming-induced bone was normalized to the non-operated limb.

To quantify the total bone volume (BV, mm^3^) in each BMP-induced bone pellet, micro Computed Tomography (microCT) scanning was performed using SkyScan 1174 compact microCT scanner (SkyScan, Kontich, Belgium). The X-ray source voltage was set at 50 kV and beam current set at 800 μA. To minimize the beam hardening artifacts a 0.5 mm aluminum X-ray beam filter was used to attenuate soft X-rays at the source. Samples were scanned at 8.7 μm resolution and 0.4° angular increments. Acquisition time was approximately 30 minutes/specimen. BV analysis was performed using CTAnalyser software, version 1.9.2.3 (SkyScan). A global grey value threshold representing new bone formation (47-255) was defined and applied to all samples. Total bone volume was calculated for the entire pellet. Representative three-dimensional bone pellets were reconstructed with transaxial slices (100 slices) of the pellet's mid-section, using CTVol Realistic Visualisation software version 2.1.0.0 (SkyScan).

For statistical analysis of *in vivo *data, group sizes <10 necessitated stringent non-parametric statistical tests. Kruskal Wallis and Mann Whitney U tests were performed using SPSS Statistics version 17 (SPSS Inc., Chicago, IL, United States).

### Histological Analysis

Samples were harvested and fixed in 4% PFA and stored in 70% ethanol. Bone was decalcified at room temperature on a shaker in 0.34 M EDTA (pH 8.0) containing 0.5% PFA for 3 days, followed by 38 days in 0.34 M EDTA (pH 8.0) with changes every 3-4 days. Samples were embedded in paraffin blocks and 5 mm-thick sections cut. Sections were stained with Picrosirius red and Alcian blue for bone and cartilage.

## Results

### *In vitro *interactions of TGF-β and BMP signaling pathways

To verify the capacity of TGF-β signaling to modulate osteogenesis we tested BMP-2, TGF-β, and the ALK-4/5/7 inhibitor SB431542 in the MC3T3-E1 pre-osteoblastic cell line that is commonly employed to model osteoblast differentiation. Without treatment, MC3T3-E1 cells express the alkaline phosphatase (AP) enzyme at day 3, and matrix mineralization was seen at day 10. Matrix mineralization in particular is an unambiguous marker of mature bone cell function. Colorimetric assays were selected to facilitate rapid screening. To further validate the colorimetric assays, qPCR markers of early osteoblast differentiation were also examined at 3 days and mature markers at 10 days.

**Figure 1:**
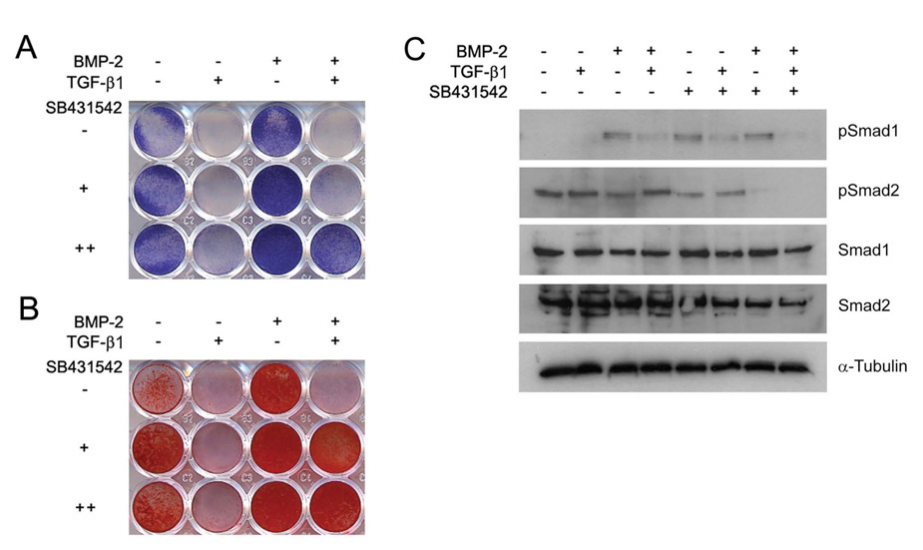
**Effects of TGF-β, BMP-2, and SB431542 on cultured MC3T3-E1 pre-osteoblasts**. Cells were grown and treated with agents (50 ng/ml BMP-2, 5 ng/ml TGF-β1, and 1 μM or 5 μM SB431542) and subjected to a rapid staining protocol that visually indicated osteogenic differentiation at 3 days (**A**, Alkaline Phosphatase) or matrix mineralization at 10 days (**B**, Alizarin Red S). These results confirmed that SB431542 can promote both early osteoblast differentiation and mature osteoblast function *in vitro*, and these results were independently repeated. To confirm mechanistically the effects of these compounds on downstream signaling (SMAD activity), Western analysis was performed on day 3 samples using pSMAD1, pSMAD2, SMAD1, SMAD2 and α-Tubulin antibodies. These data indicated that BMP-2 and SB431542 could promote SMAD1 phosphorylation and reduce SMAD2 phosphorylation.

Dose response curves were performed for individual agents (data not shown) and doses that showed sub-maximal and no toxic effects when given individually were selected for further analysis in combination. As expected, AP (Figure 1A) and mineralization stains (Figure 1B) were suppressed by exogenous TGF-β1 treatment and augmented with BMP-2 treatment. The SB431542 inhibitor was found to be sufficient to promote osteogenic differentiation in isolation and was able to produce additive effects when co-treated with BMP-2. Neither SB431542 nor BMP-2 could individually overcome the repressive effects of TGF-β1 on osteogenesis, although this was observed when they were used in combination. The comparable outcomes seen for AP and mineralization indicate these agents do not have differing effects on early osteoblast differentiation and mature osteoblast function.

In a subsequent experiment, cDNA was synthesized from treated cells and this was analyzed by qPCR. Bone genes included the early osteogenic/chondrogenic commitment factor *Runx2 *(Table 1A), the osteoblastic marker *Alp *(Table 1B), and the mature bone marker *osteocalcin (Ocn) *(Table 1C). Gene expression profiles reflected the AP and mineralization staining data results with SB431542 able to augment osteogenesis alone and in combination with BMP-2. These data reinforce the concept that blockade of the ALK-4/5/7 receptors can augment the differentiation of committed osteoprogenitors *in vitro*.

**Table 1:** *Runx2, Alp and Ocn *expressions in day 3 samples (fold-change in gene expression)

(A) *Runx2 *expression in day 3 samples (fold-change in gene expression)		
	0 μM SB431542	5 μM SB431542
no BMP-2/TGF-β1	1.00 (0.11)	1.77 (0.15)
50 ng/ml BMP-2	1.62 (0.10)	3.59 (0.34)
5 ng/ml TGF-β1	0.54 (0.10)	2.01 (0.09)
50 ng/ml BMP-2, 5 ng/ml TGF-β1	0.56 (0.12)	2.05 (0.23)
		
**(B) *Alp *expression in day 3 samples (fold-change in gene expression)**		
	**0 μM SB431542**	**5 μM SB431542**

no BMP-2/TGF-β1	1.00 (0.13)	3.51 (0.25)
50 ng/ml BMP-2	5.50 (1.25)	11.05 (0.29)
5 ng/ml TGF-β1	0.15 (0.04)	0.92 (0.08)
50 ng/ml BMP-2, 5 ng/ml TGF-β1	0.27 (0.05)	3.47 (0.38)
		
**(C) *Ocn *expression in day 10 samples (fold-change in gene expression)**		
	**0 μM SB431542**	**5 μM SB431542**

no BMP-2/TGF-β1	1.00 (0.06)	1.45 (0.25)
50 ng/ml BMP-2	4.11 (0.28)	5.18 (0.34)
5 ng/ml TGF-β1	0.06 (0.01)	0.70 (0.03)
50 ng/ml BMP-2, 5 ng/ml TGF-β1	0.69 (0.06)	3.72 (0.06)

### Modulation of R-SMAD activity

While BMP and TGF-β can mediate strong transient changes in SMAD activity, particularly in serum starved cells, we sought to determine the sustained activation of SMADs concomitant with osteogenic differentiation as has been examined previously [23]. The phosphorylation status of SMAD1 and SMAD2 were assayed as representative of the activity of the osteogenic (BMP-stimulated) and non-osteogenic (TGF-β1 stimulated) R-SMADs respectively. Consistent with the onset of AP expression, samples were collected at day 3. The phosphorylated (pSMAD1, pSMAD2) forms of the protein were examined by western blotting, with total SMAD1 and α-Tubulin included as a loading controls (Figure 1C). BMP-2 increased pSMAD1 levels, and also reduced pSMAD2 levels. While the classical model for TGF-β1 signaling indicates that it acts primarily via pSMAD2, we observed in the MC3T3-E1 system that exogenous TGF-β1 treatment suppressed pSMAD1 and that pSMAD2 levels were unchanged. SB431542 both reduced pSMAD2 and increased pSMAD1, suggesting a sophisticated signaling mechanism for this compound.

### Effects of SB431542 in a murine marrow ablation model

Healthy C57BL6/J mice underwent a surgical procedure where the marrow cavity was reamed to promote fibrosis followed by new bone formation. An *in vivo *dose of 1 mg/kg/day SB431542 was initially extrapolated from the effective 2 μg/ml (5 μM) *in vitro *dose based on the ratio of systemic PTH_1-34 _(25 μg/kg/wk) to an effective *in vitro *dose of 50 ng/ml known to stimulate primary osteoblast differentiation (data not shown).

**Figure 2:**
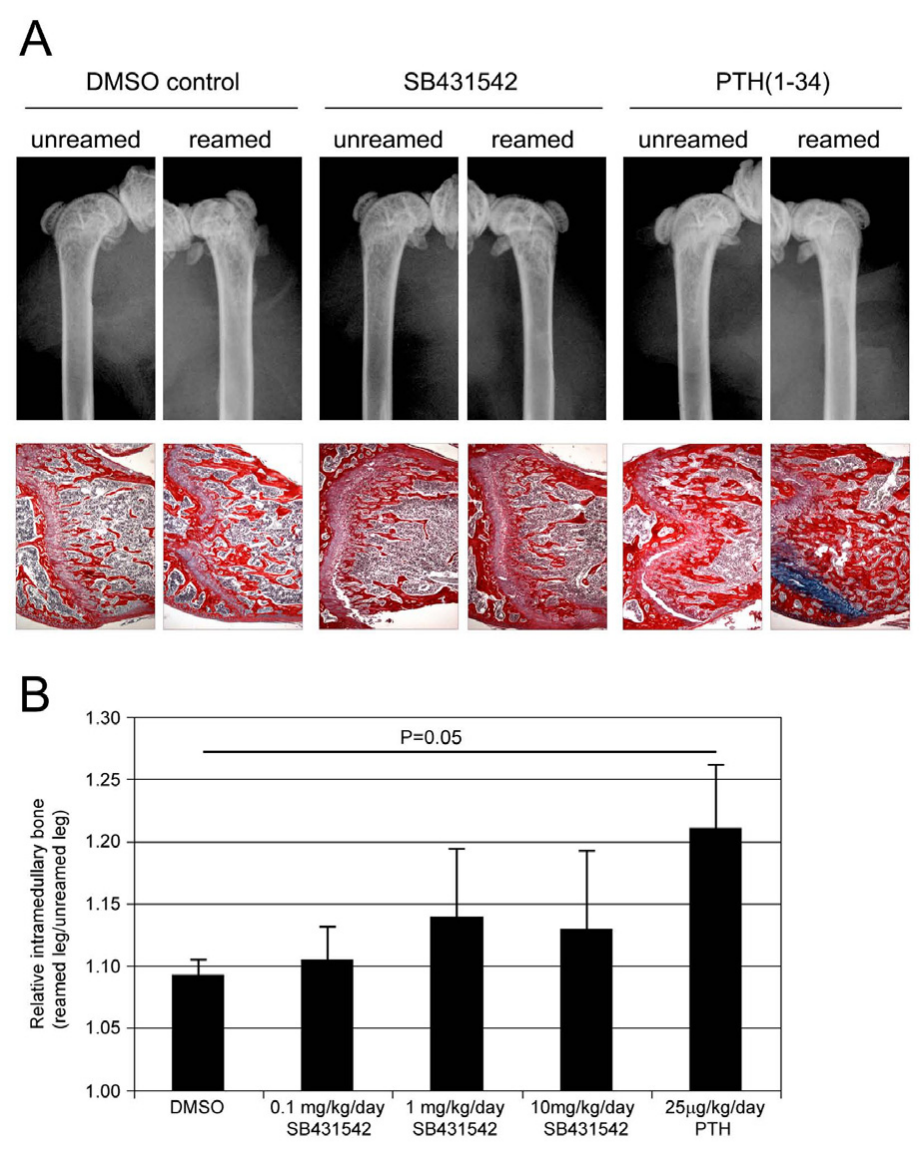
**Radiographic analysis of the marrow ablation model**. This model utilizes femoral reaming to induce new bone formation with femora harvested at day 10. Representative X-rays and histological sections (**A**) of DMSO vehicle, 1 mg/kg/day SB431542, and 25 μg/kg/day PTH_1-34 _treated groups are shown. Radiographs illustrate an overall increase an intramedullary bone with reaming throughout the operated limb, particularly with PTH_1-34 _treatment. This is also seen below the growth plate (oriented to the left) in histological sections with bone stained with Picrosirius red. To test and quantify the effects of a range of inhibitor doses in this model, DMSO vehicle, 0.1-10 mg/kg/day SB431542, and 25 μg/kg/day PTH_1-34 _treated groups were analyzed by pQCT (**B**). The marrow ablation-induced bone was analyzed by calculating the average BMC of reamed limbs relative to unreamed limbs. Only PTH_1-34 _treatment created a significant increase in reaming-induced bone formation (P = 0.05).

In an initial screen to validate the model system, mice were given DMSO vehicle alone, 25 μg/kg/day PTH_(1-34)_, or 1 mg/kg/day SB431542 with all drugs given s.c. Hind limbs were harvested after 10 days and analyzed by X-ray, histology, and pQCT scanning. X-ray images showed the presence of additional mineralized tissue within the intramedullary canals of reamed limbs, indicative of new bone formation (Figure 2A). Quantification by pQCT showed consistent increases in BMC in the reamed limbs compared to the non-operated limbs of DMSO treated (P = 0.055), SB431542 treated (P = 0.055), and PTH_(1-34) _treated (P = 0.004) animals. These differences were more evident in terms of new bone directly below the growth plate in histological sections (Figure 2A). Nevertheless, neither pQCT nor histology showed any additional improvement in marrow ablation-induced new bone formation with SB431542 treatment (12% decrease in BMC with SB431542 over DMSO, P = 0.26). In contrast, PTH_(1-34) _treatment greatly increased bone formation in the reamed limbs (P = 0.055), and this was also seen histologically (Figure 2A). The strong effects seen with PTH_(1-34) _validated the capacity of this model to reveal the effects of potent bone anabolics.

In a second experiment a wider range of SB431542 doses were used (0.1-10 mg/kg/day). There was concern that doses higher than 10 mg/kg/day could have toxic or non-specific effects, as *in vitro *doses above 20 μM rapidly induced cell death. For the analysis, to reduce the confounding impact of intra-mouse variation in femoral BMC, the BMC in the marrow ablated limb was calculated relative to the non-operated (unreamed) limb. Again, a consistent increase was seen in BMC with marrow ablation, although none of the SB431542 treated groups showed a significant increase in BMC compared to DMSO controls (Figure 2B). SB431542 treatment did not have an effect on BMC in the non-operated limb.

### Effects of SB431542 in a BMP-2 induced bone formation model

As an alternative method for inducing bone formation, PDLLA polymer pellets containing BMP-2 were implanted intramuscularly in quadriceps of C57BL6/J mice. In addition to the BMP-only control group, two other groups were supplemented with low dose (92 μg) and high dose (384 μg) SB431542. The BMP-2 doses were based on previous experience with the model system [25] and local SB431542 doses were extrapolated from the ratio of BMP-2:SB431542 used *in vitro*. Bone formation occurred over 3 weeks. Two mice were excluded at the experimental endpoint as misplacement or shifting of the pellet had led to the ectopic bone fusing with the femur.

**Figure 3:**
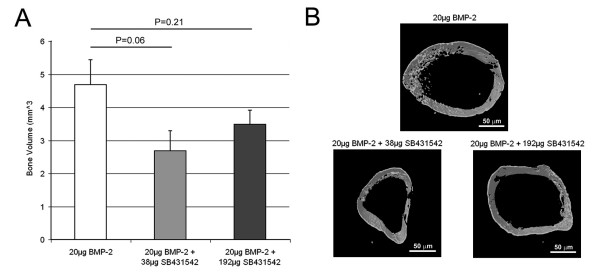
**Radiographic analysis of the BMP-2 implantation model**. This model uses 20 μg BMP-2 implanted into the hind limb to induce ectopic bone formation in 3 weeks. The effects of local SB431542 co-delivery were quantified by microCT measurement of bone volume in the entire bone pellets (**A**). Relative to BMP-2 alone, SB431542 did not increase bone formation (47% decrease, P < 0.06 for 92 μg SB431542; 25% decrease, P < 0.21 for 384 μg SB431542). This is further illustrated by representative microCT reconstructions of bone nodule cross-sections.

The bone volume of the entire pellet was visualized and quantified by microCT. The low dose SB431542 group showed a 47% reduction in bone volume compared to the BMP-only control group (P = 0.06). The higher dose SB431542 group was comparable to the BMP-only group (P = 0.21) (Figure 3A). Neither group supported the initial hypothesis that SB431542 could increase BMP-2 induced bone formation. For representative pellets, transaxial sections in the centers of the pellets were reconstructed to create 3 D models (Figure 3B).

## Discussion

The study by Maeda *et al*. (2004) used an *in vitro *system to characterize the response of myogenic progenitors to BMP signaling and treatment with SB431542 [23]. They showed that SB431542 enhanced the effects of BMP-2 on osteogenesis and that this was associated with increased SMAD1 signaling and decreased SMAD2 signaling. Our data using the MC3T3-E1 cell line supports a pro-osteogenic effect of SB431542 on pre-osteoblasts, even in the absence of exogenous BMP-2. In terms of a screening system for novel compounds, the MC3T3-E1 system is rapid, low-cost, and suitable for generating rapid dose response curves. Due to limitations with this cell line [30,31], prospective agents should also be trialed on primary mesenchymal stem cells, however in the case of SB431542 this data was already available [23]. Maeda *et al *also examined the expression of I-SMADs (inhibitory SMADs), which are downstream negative regulators of R-SMAD signaling, and showed a suppression of SMAD6 and SMAD7 by SB431542 with prolonged treatment [23]. While I-SMADs represent potentially important modifiers of R-SMAD signaling, they are transcriptionally regulated by and secondary to the initial R-SMAD response. Our *in vitro *data indicates that BMP and TGF-β signals can modulate R-SMAD signaling in a non-canonical fashion. Specifically, ALK-4/5/7 inhibition led to increases in pSMAD1 levels and BMP-2 treatment led to a reduction in pSMAD2 levels (Figure 1).

In this study we have also employed two rapid surgical models to screen for pro-osteogenic effects in a bone formation/bone repair context. The first was a marrow ablation model previously described in the context of biglycan null mice that show decreased bone formation following reaming [29]. We adopted a high-resolution multi-slice pQCT scanning approach for quantification, which was found to give more accurate results than individual sections that were more susceptible to positional effects. We confirmed that reaming consistently induced new bone formation in all groups. PTH_(1-34) _administration further increased the amount of bone on the reamed side, validating the model system. In contrast, SB431542 did not produce any substantive pro-osteogenic effect in reamed bones or in non-operated limbs.

The second model was a BMP-2 intramuscular implantation model [25], which contains an endochondral bone formation component. Again, no significant increase was observed in bone formation with SB431542 treatment, rather a trend was seen towards reduced bone with local dosing (Figure 3).

The lack of a beneficial effect of the TGF-β inhibitor SB431542 in the *in vivo *models may be due to several reasons. One possibility was an inappropriate dose selection, although higher doses were likely to non-specifically affect other receptors. In a previously published study, a single dose of 0.2 mg/kg was used to affect metabolic changes in rats [32], indicating that our dose range of up to 10 mg/kg/day should be capable of producing significant physiological effects in mice. This SB431542 compound has also been successfully used in organ culture experiments to produce developmental effects [33]. Nevertheless, the specificity and/or bioavailability of SB431542 may be suboptimal for *in vivo *studies, and there certainly exists the potential for more specific inhibitor compounds to produce improved results.

An alternative explanation for the disparity between *in vitro *and *in vivo *results may be due to the fundamental differences between the techniques and outcome measures in the different systems. Cell culture models focus primarily on the process of cell differentiation, generally on committed bone cells [11-14,23]. In contrast, surgical models also incorporate elements of osteoprogenitor recruitment and proliferation. In the context of TGF-β, this may be critical as TGF-β release has been recently shown to play a major role in the recruitment of osteoprogenitors for bone homeostasis [34]. Thus our study may highlight a basic limitation of *in vitro *systems and stress the utility of expediting screens with surgical models such as the marrow ablation or BMP-2 implantation model.

## Conclusions

Our data confirms that TGF-β inhibition can enhance the differentiation of committed osteoprogenitors in culture, and these effects were additive with BMP-2 treatment. However, these cell culture phenomena did not translate into increased bone formation in a marrow ablation or BMP-induced ectopic bone models. This may be due to effects of TGF-β on osteoprogenitor recruitment that are not modeled *in vitro*. Findings from marrow ablation and BMP-2 implantation models suggest that sustained global TGF-β suppression with SB431542 is likely to be ineffective for orthopaedic applications.

## Competing interests

The authors declare that they have no competing interests.

## Authors' contributions

**AS **conceptualized and organized the study. AM performed cell culture and histology analyses with the aid of RL, SK, and MMD. LP and KM performed surgical experiments in mice. NYCY performed quantative microCT analysis with support by PAB and AJR. DGL was the senior researcher who guided the research study. The study was primarily written by AS and DGL, although all authors read and approved the manuscript.

## Pre-publication history

The pre-publication history for this paper can be accessed here:

http://www.biomedcentral.com/1471-2474/11/105/prepub
